# A novel computational model for predicting potential LncRNA-disease associations based on both direct and indirect features of LncRNA-disease pairs

**DOI:** 10.1186/s12859-020-03906-7

**Published:** 2020-12-02

**Authors:** Yubin Xiao, Zheng Xiao, Xiang Feng, Zhiping Chen, Linai Kuang, Lei Wang

**Affiliations:** 1 College of Computer Engineering and Applied Mathematics, Changsha University, Changsha, 410001 People’s Republic of China; 2 Hunan Province Key Laboratory of Tumor Cellular and Molecular Pathology, Cancer Research Institute, University of South China, Hengyang, 421001 Hunan People’s Republic of China; 3 Key Laboratory of Hunan Province for Internet of Things and Information Security, Xiangtan University, Xiangtan, 411105 People’s Republic of China

**Keywords:** LncRNA-disease association prediction, Features, Random walk, Multiple linear regression, Artificial neural network

## Abstract

**Background:**

Accumulating evidence has demonstrated that long non-coding RNAs (lncRNAs) are closely associated with human diseases, and it is useful for the diagnosis and treatment of diseases to get the relationships between lncRNAs and diseases. Due to the high costs and time complexity of traditional bio-experiments, in recent years, more and more computational methods have been proposed by researchers to infer potential lncRNA-disease associations. However, there exist all kinds of limitations in these state-of-the-art prediction methods as well.

**Results:**

In this manuscript, a novel computational model named FVTLDA is proposed to infer potential lncRNA-disease associations. In FVTLDA, its major novelty lies in the integration of direct and indirect features related to lncRNA-disease associations such as the feature vectors of lncRNA-disease pairs and their corresponding association probability fractions, which guarantees that FVTLDA can be utilized to predict diseases without known related-lncRNAs and lncRNAs without known related-diseases. Moreover, FVTLDA neither relies solely on known lncRNA-disease nor requires any negative samples, which guarantee that it can infer potential lncRNA-disease associations more equitably and effectively than traditional state-of-the-art prediction methods. Additionally, to avoid the limitations of single model prediction techniques, we combine FVTLDA with the Multiple Linear Regression (MLR) and the Artificial Neural Network (ANN) for data analysis respectively. Simulation experiment results show that FVTLDA with MLR can achieve reliable AUCs of 0.8909, 0.8936 and 0.8970 in 5-Fold Cross Validation (fivefold CV), 10-Fold Cross Validation (tenfold CV) and Leave-One-Out Cross Validation (LOOCV), separately, while FVTLDA with ANN can achieve reliable AUCs of 0.8766, 0.8830 and 0.8807 in fivefold CV, tenfold CV, and LOOCV respectively. Furthermore, in case studies of gastric cancer, leukemia and lung cancer, experiment results show that there are 8, 8 and 8 out of top 10 candidate lncRNAs predicted by FVTLDA with MLR, and 8, 7 and 8 out of top 10 candidate lncRNAs predicted by FVTLDA with ANN, having been verified by recent literature. Comparing with the representative prediction model of KATZLDA, comparison results illustrate that FVTLDA with MLR and FVTLDA with ANN can achieve the average case study contrast scores of 0.8429 and 0.8515 respectively, which are both notably higher than the average case study contrast score of 0.6375 achieved by KATZLDA.

**Conclusion:**

The simulation results show that FVTLDA has good prediction performance, which is a good supplement to future bioinformatics research.

## Background

LncRNAs have long been considered as a transcriptional noise [1, 2]. However, in recent years, more and more researches have shown that lncRNAs play key roles in numerous important biological processes of humans, including chromatin modification, epigenetic regulation, cell cycle control, cell differentiation and so on [3–6]. Especially, accumulating bio-experiments have confirmed that mutations and dysregulation of lncRNAs are associated with the development of diseases, such as leukemia [7], neurological disorders [8], coronary artery diseases [9] and several cancers [10]. Hence, effectively inferring potential associations between lncRNAs and diseases can not only contribute to understand the pathogenesis of some complex diseases at the molecular level, but also be conducive to provide biomarkers for disease diagnosis, therapy and prognosis. Up to now, along with the rapid increment of newly inferred lncRNAs, some publicly available lncRNA-related databases, including lncRNADisease [11], NONCODE [12], lncRNAdb [13] and NRED [14], have been established successively. However, the number of known lncRNA-disease associations is still very limited, since traditional biological experiments are costly and time-consuming. Therefore, it is important and necessary to construct effective and high-throughput computational models to explore potential lncRNA-disease associations.

So far, researchers have developed numerous powerful computational models to predict potential lncRNA-disease associations, which can be roughly classified into three major categories according to their main implementation strategies [15]. Among them, the first category aims to adopt machine learning methods to predict potential lncRNA-disease associations. For example, Yu and Wang et al. proposed a prediction model based on the Naïve Bayes classifier [16] in 2018 and a prediction model based on the collaborative filtering algorithm [17] in 2019 to infer potential lncRNA-disease associations, respectively. Xuan and Wang et al. developed a probabilistic matrix factorization model based on the semi-supervised learning method to identify potential associations between lncRNAs and diseases [18]. In these prediction models of the first category, the major drawback lies in the requirement of negative samples as the training set, which will affect their prediction performances notably, since the negative samples are usually difficult to obtain. Of course, some models overcome this limitation. LRLSLDA is the first large-scale prediction model [19], which does not need the negative samples information, but how to choose the best parameters remains to be solved.

Different from the first category, the second category focuses on implementing propagation algorithms such as Random Walk on a heterogeneous network constructed by integrating lncRNA-disease association network, disease similarity network and lncRNA similar network, etc. For instance, in 2014, Sun et al. [20] established a global network-based computational model, which adopted the random walk with restart (RWR) algorithm to predict potential lncRNA-disease associations. In 2015, Zhou et al. [21] proposed a prediction model by implementing RWR on a heterogeneous network comprising known lncRNA-disease association network, miRNA-associated lncRNA crosstalk network and disease similarity network. However, these two models mentioned above can only be applied to infer lncRNAs with related-disease or known miRNA-disease associations. To break through this kind of limitation, in 2015, Chen et al. [22] developed a computational model called KATZLDA for prediction of potential lncRNA-disease associations, which can infer potential lncRNAs in the absence of known associated diseases. But prediction may bias in favor of lncRNAs with more known related-diseases and diseases with more known related-lncRNAs as well due to its construction of the network.

According to the above descriptions, the prediction performance of all these models of both categories will be influenced by the number of known lncRNA-disease associations. However, the number of known lncRNA-disease associations confirmed by bio-experiments is still very limited. Therefore, to avoid the drawback of limited known lncRNA-disease associations, the third category adopts indirect biological information to explore the prediction of potential lncRNA-disease associations. For instance, in 2014, Liu et al. [23] proposed a novel prediction model by combining human lncRNA expression profiles, human disease-associated gene data and gene expression profiles, which can achieve exciting prediction performance while there are no known lncRNA-disease associations. However, it cannot implements to predict lncRNAs without gene-related records.

Different from the above existing methods, in this manuscript, we proposed a novel computational model named FVTLDA to reveal potential lncRNA-disease associations. In FVTLDA, to avoid the limitation of various methods mentioned previously, we first introduce direct and indirect biological information on lncRNAs and diseases, including known lncRNA-miRNA-disease associations. Then, known lncRNA-disease associations will be utilized to extract direct features for lncRNA-disease pairs based on the concept of Disease Clique. Meanwhile, indirect biological information including known miRNA-disease associations and known miRNA-lncRNA associations will be utilized to extract indirect features for lncRNA-disease pairs by adopting the random walk with restart. What's more, to avoid the limitation of single model prediction techniques, based on the direct and indirect features obtained for lncRNA-disease pairs, the Multiple Linear Regression (MLR) and Artificial Neural Network (ANN) will be combined with FVTLDA to reveal potential lncRNA-disease associations, respectively. To estimate the prediction performance of FVTLDA, different frameworks including the LOOCV, fivefold CV and tenfold CV are implemented to compare FVTLDA with existing competing models. Simulation experiment results show that FVTLDA with MLR can achieve AUCs of 0.8909, 0.8936 and 0.8970 in fivefold CV, tenfold CV and LOOCV respectively, while FVTLDA with ANN can achieve AUCs of 0.8766, 0.8830 and 0.8807 in fivefold CV, tenfold CV and LOOCV separately, which both outperform existing state-of-the-art models. Meanwhile, in case studies of gastric cancer, leukemia and lung cancer, simulation experiment results show that there are 8, 8 and 8 out of top 10 candidate lncRNAs predicted by FVTLDA with MLR, and 8, 7 and 8 out of top 10 candidate lncRNAs predicted by FVTLDA with ANN, having been verified respectively in biological experimental studies or other independent studies. Finally, to further illustrate actual predictive ability of FVTLDA, we have compared it with the representative prediction model KATZLDA based on the new concept of case study contrast score as well, which aims to quantify the prediction ability of the model in case study. And simulation experiment results show that the average case study contrast scores of FVTLDA with MLR and FVTLDA with ANN are 0.8429 and 0.8515 respectively, which both outperform the average case study contrast score of 0.6375 obtained by KATZLDA notably.

## Result

### Performance evaluation

In order to evaluate the prediction performance of FVTLDA, in this section, we implement the LOOCV on FVTLDA as follows: For all known lncRNA-disease pairs, each pair with known correlations was selected in turn for testing, and other lncRNA-disease pairs were retained as training samples for model learning. Particularly, testing samples and lncRNA-disease pairs without known correlations were considered as candidates. After the implementation of FVTLDA, the ranking positions of test samples in candidates can be obtained according to the association probability fractions. If the ranking of a test sample is above the given threshold, it will be seen as a successful prediction or a positive sample. Otherwise, it is seen as an unsuccessful prediction or a negative sample. Besides, upon different thresholds, the corresponding true positive rate (TPR, sensitivity) and false positive rate (FPR, 1 − specificity) can be calculated as follows:

1 $$TPR = \frac{TP}{{TP + FN}}$$

2 $$FPR = \frac{FP}{{TN + FP}}$$

Here, *TP* and *TN* represent the correctly identified positive and negative samples separately, while FP and FN denote the incorrectly identified positive and negative samples, respectively.

Based on the above equations, the Receiver Operating Characteristic (ROC) curve can be drawn according to the TPRs and FPRs of different thresholds, and the area under ROC curve (AUC) will further be calculated to evaluate the performance of FVTLDA. The AUC value of 1 indicates the perfect prediction performance while the AUC value of 0.5 means a random guess.

**Fig. 1:**
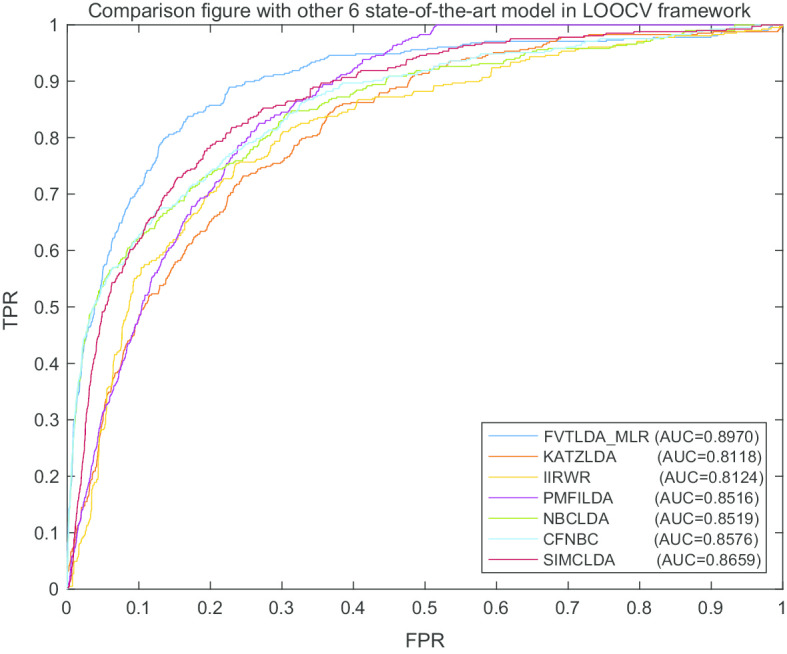
The AUCs achieved by FVTLDA_MLR, KATZLDA, IIRWR, PMFILDA, NBCLDA, CFNBC and SIMCLDA in framework of LOOCV

During simulation, we first compared FVTLDA_MLR (i.e., FVTLDA with MLR) with six state-of-the-art prediction models such as NBCLDA [16], CFNBC [17], PMFILDA [18], KATZLDA [22], SIMCLDA [24] and IIRWR [25] in the framework of LOOCV, and comparison results were shown in Fig. 1. Through observing this figure, it can be seen that FVTLDA_MLR can achieve AUC of 0.8970, which significantly outperforms those six state-of-the-art prediction models with the increment of AUC values by at least 0.0311.

Moreover, to eliminate the random error caused by the random initialization of weights and biases in FVTLDA_ANN (i.e., FVTLDA with ANN), during simulation, we repeated the execution of LOOCV on FVTLDA_ANN for 20 times, and took the mean and variance of the AUC values as the result. As illustrated in Additional file 1, it can be seen that FVTLDA_ANN achieves a reliable mean of AUC value of 0.8807 and standard deviation (std) of 0.0047 in LOOCV, which outperforms these six state-of-the-art prediction models.

**Fig. 2:**
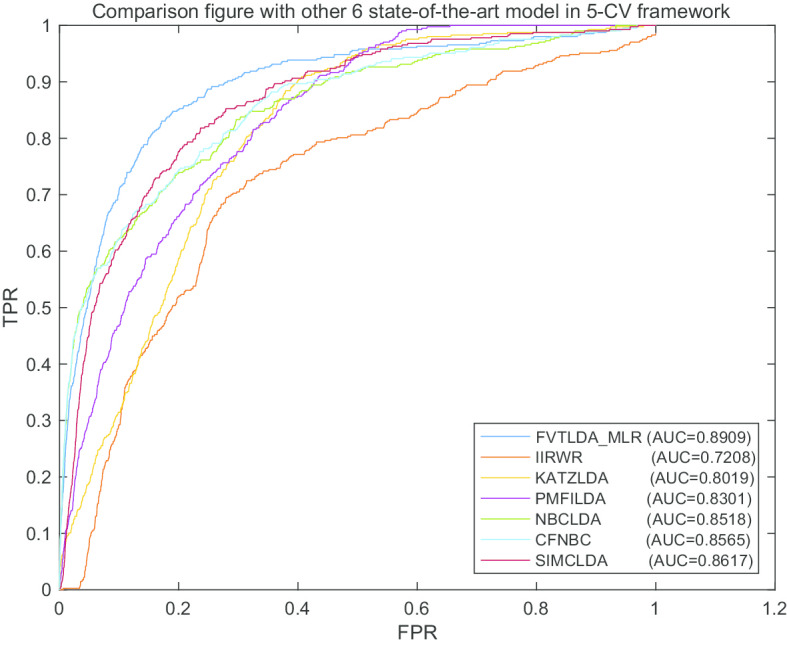
The AUCs achieved by FVTLDA_MLR, KATZLDA, IIRWR, PMFILDA, NBCLDA, CFNBC and SIMCLDA in framework of fivefold CV

**Fig. 3:**
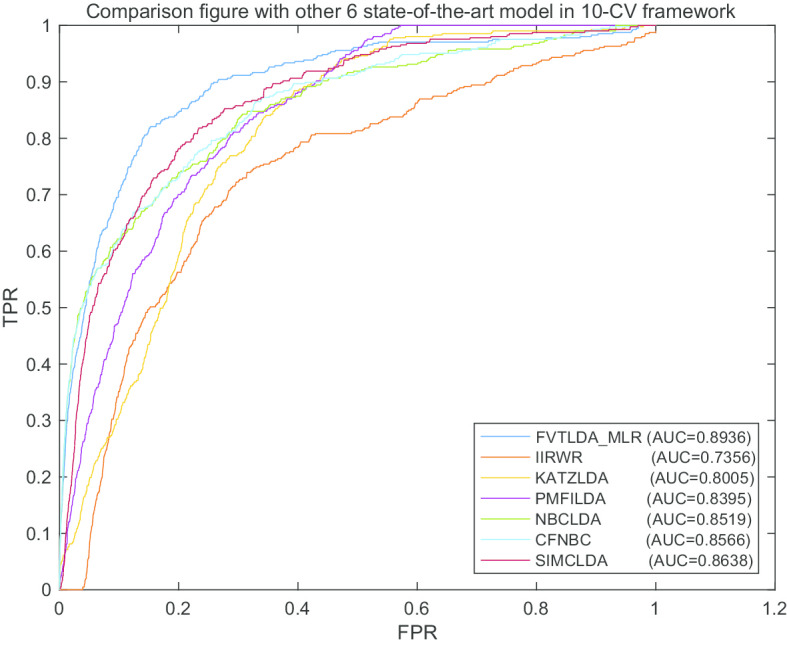
The AUCs achieved by FVTLDA_MLR, KATZLDA, IIRWR, PMFILDA, NBCLDA, CFNBC and SIMCLDA in framework of tenfold CV

In order to further verify the prediction performance of FVTLDA while there are few known lncRNA-disease associations, the frameworks of *K*-fold CV including fivefold CV and tenfold CV were implemented to compare FVTLDA_MLR with other representative prediction models. During implementing the *K*-fold CV, all known lncRNA-disease associations are equally divided into *K* parts, each part was left out as the test sample in turn, and other remaining lncRNA-disease pairs were used as the training samples. As shown in the following Figs. 2 and 3, FVTLDA_MLR can achieve better predictive performance than the other six competing models, which demonstrates that FVTLDA can perform better in sparse data sets as well.

Furthermore, in order to eliminate the effects of the random partition of training samples, during simulation, we repeated the implementations of 5-CV and 10-CV 20 times respectively, and took the mean and variance of AUC value as the results. As shown in Additional files 2 and 3, FVTLDA_MLR achieves the mean AUC value of 0.8903 and std of 0.0022 in 5-CV, and the mean AUC of 0.8940 and std of 0.0014 in 10-CV, separately. Meanwhile, as for FVTLDA_ANN, from observing the following Additional files 4 and 5, it can be seen that it achieves the mean AUC value of 0.8766 and std of 0.0043 in 5-CV, and the mean AUC of 0.8830 and std of 0.0022 in 10-CV, respectively.

**Fig. 4:**
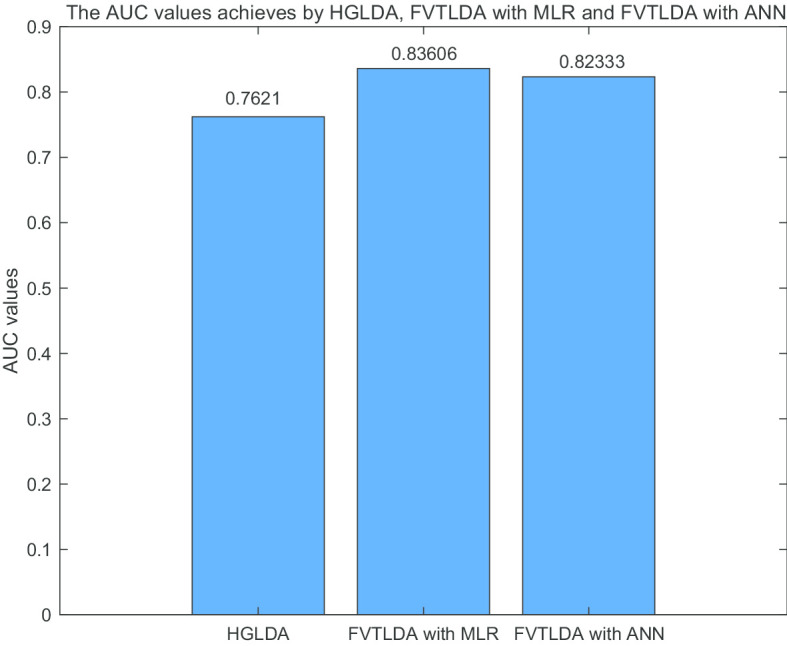
The AUC values achieved by FVTLDA_MLR, FVTLDA_ANN and HGLDA

**Fig. 5:**
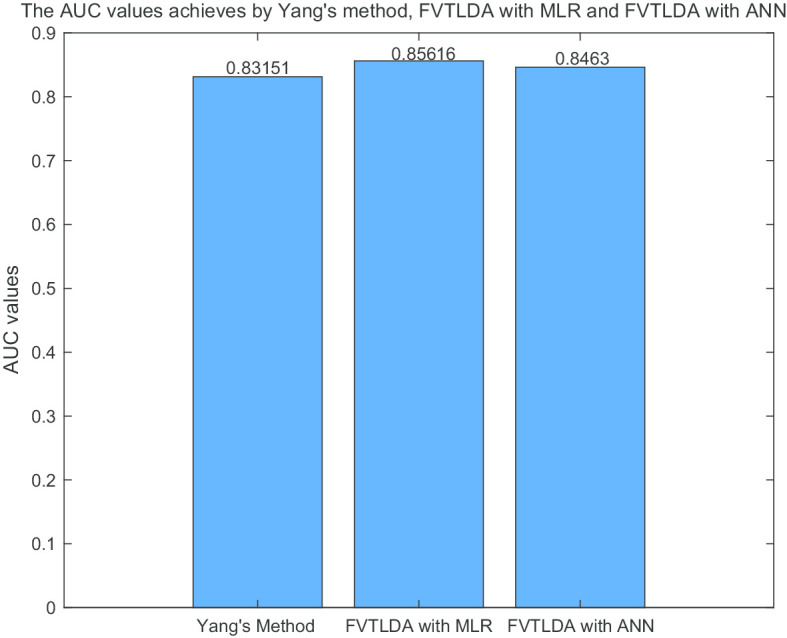
The AUC values achieved by FVTLDA_MLR, FVTLDA_ANN and Yang’s method

Finally, to demonstrate that FVTLDA can perform well in different data sets, we further compared it with other state-of-the-art models including HGLDA [26] and the method proposed by Yang et al. [27] in the framework of LOOCV. While comparing FVTLDA with HGLDA, we adopted the data set given by HGLDA, which consists of 183 experimentally validated lncRNA-disease associations. While comparing FVTLDA with the method proposed by Yang et al., we used the dataset put forward by Yang et al., which consists of 319 known lncRNA-disease associations. FVTLDA outperforms these two kinds of model in different datasets (Figs. 4 and 5).

### Parameter analysis

In this section, influences of parameters in FVTLDA are estimated. The parameters *r*_1_ and *r*_2_ in Eq. (11) (See the section of Methods) and Eq. (14) represent the restart probabilities of the random walk, the parameter *rate* in Eq. (19) stands for the adjustment factor, and the parameters *k*_1_ and *k*_2_ in Eqs. (20) and (21) denote the attenuation factors, respectively.

In order to determine the optimal values of the above five parameters efficiently, we traverse the approximate range of each parameter through FVTLDA with MLR in the framework of LOOCV (0, 0.0001, 0.001, 0.01, 0.1). For parameters that can further improve the precision, we take the approximate solution of the previous step as the default value, and then, the optimal solution with higher precision is achieved by traversal. As illustrated in the following Table 1 (bold represents the best parameter), the optimal values for these five parameters such as *rate*, *r*_1_, *r*_2_, *k*_1_, and *k*_2_ are 0.3, 0.001, 0.001, 0.008, 0.007 separately.

**Table 1:** Effects of the parameter to the performance of FVTLDA_MLR in LOOCV

Rate	0	0.1	0.2	0.3	0.4	0.5	0.6	0.7	0.8	0.9	1
AUC	0.89698	0.89699	0.89699	0.89701	0.89697	0.89695	0.89696	0.89697	0.89695	0.89694	0.89693
*r*_1_	0	0.001	0.002	0.003	0.004	0.005	0.006	0.007	0.008	0.009	0.01
AUC	0.72933	0.89701	0.89694	0.89689	0.89678	0.89673	0.89671	0.89668	0.89665	0.8966	0.89647
*r*_2_	0	0.001	0.002	0.003	0.004	0.005	0.006	0.007	0.008	0.009	0.01
AUC	0.79486	0.89701	0.897	0.89692	0.89692	0.89693	0.89692	0.89693	0.89684	0.8968	0.89647
*k*_1_	0	0.001	0.002	0.003	0.004	0.005	0.006	0.007	0.008	0.009	0.01
AUC	0.89699	0.89701	0.897	0.897	0.89698	0.89695	0.89698	0.8967	0.89701	0.897	0.89699
*k*_2_	0	0.001	0.002	0.003	0.004	0.005	0.006	0.007	0.008	0.009	0.01
AUC	0.89698	0.89699	0.897	0.89699	0.897	0.897	0.897	0.89701	0.89699	0.89697	0.89698

### Case study

In order to further demonstrate the predictive ability of FVTLDA, in this section, we select gastric cancer, leukemia and lung cancer as case studies. During the simulation, for any given disease *d*_*i*_ $\in${the gastric cancer, the leukemia, the lung cancer}, only those lncRNAs that do not have known associations with *d*_*i*_ will be considered as validated candidates for *d*_*i*_. Next, all candidate lncRNAs will be ranked according to their association probability fractions calculated by FVTLDA. Finally, the top 10 candidate *d*_*i*_-related lncRNAs will be verified by recent articles and experiments published in the NCBI database (https://www.ncbi.nlm.nih.gov/). Additionally, to compare the difference of prediction performance between FVTLDA_MLR and FVTLDA_ANN, as well as the difference of prediction performance between FVTLDA and another representative prediction model KATZLDA, we further list all these lncRNAs in the top 10 candidate *d*_*i*_-related lncRNAs predicted by FVTLDA_MLR, FVTLDA_ANN and KATZLDA separately. Simultaneously, we will provide corresponding rankings and relevant evidence of these lncRNAs as well. Moreover, in order to visualize the predictive ability of these three kinds of prediction models in the above case studies, we propose a novel concept of case study contrast score, which can be calculated as follows:

3 $$score = \exp \left( {\mathop \sum \limits_{i = 1}^{m} \frac{1}{{R_{i} }} - \frac{1}{i}} \right)$$

Here, *m* denotes the number of verified lncRNAs in top 10 predicted candidate lncRNAs, and *R*_*i*_ represents the ranking corresponding to the *i*th confirmed lncRNA. If the model has better practical ability, the closer the score of the model is to 1. For example, in Table 2, the case study contrast score of FVTLDA_MLR = $e^{{\left( {1 + \frac{1}{2} + \frac{1}{4} + \frac{1}{5} + \cdots + \frac{1}{7} + \frac{1}{9} + \frac{1}{10} + \frac{1}{29} + \frac{1}{11}} \right) - \left( {1 + \frac{1}{2} + \frac{1}{3} + \cdots + \frac{1}{9} + \frac{1}{10}} \right)}} = 0.7168$.

**Table 2:** Top 10 potential gastric cancer-related lncRNAs and their PubMed unique identifiers predicted by FVTLDA_MLR, FVTLDA_ANN and KATZLDA

lncRNA	Evidence (PMID)	Rank by FVTLDA_MLR	Rank by FVTLDA_ANN	Rank by KATZLDA
MALAT1	29,719,612	1	1	1
PVT1	25,258,543	2	3	2
HOTAIRM1	Unknown	3	11	15
GAS5	29,557,411	4	2	3
TUG1	29,719,612	5	4	4
NEAT1	28,401,449	6	7	5
XIST	27,620,004	7	5	6
KCNQ1OT1	Unknown	8	6	8
HOXA11-AS	28,441,948	9	13	27
MIAT	29,540,201	10	10	7
SNHG7	29,131,253	29	8	34
TP73-AS1	Unknown	17	9	35
ZNRD1-AS1	Unknown	12	60	9
HOTTIP	27,144,338	11	42	10

Gastric cancer is the second leading cause of cancer-related deaths and the fourth most common cancer in the world [28, 29]. Up to now, there is a large number of lncRNAs having been proved to be related to gastric cancer [30, 31]. FVTLDA_MLR, FVTLDA_ANN and KATZLDA can successfully predict 8, 8 and 8 confirmed lncRNAs out of the top 10 candidate lncRNAs respectively (Table 2), and their corresponding case study contrast scores are 0.7168, 0.8377 and 0.8439 separately.

As for leukemia, its association with some lncRNAs has been widely reported [32, 33]. FVTLDA_MLR, FVTLDA_ANN, and KATZLDA can successfully predict 8, 8 and 8 confirmed lncRNAs out of the top 10 candidates lncRNAs separately (Table 3), and their corresponding case study contrast scores are 0.9448, 0.9753 and 0.9688 respectively.

**Table 3:** Top 10 potential leukemia-related lncRNAs and their PubMed unique identifiers predicted by FVTLDA_MLR, FVTLDA_ANN and KATZLDA

lncRNA	Evidence (PMID)	Rank by FVTLDA_MLR	Rank by FVTLDA_ANN	Rank by KATZLDA
H19	15,645,136	1	1	1
MALAT1	28,713,913	2	4	3
HOTAIR	26,622,861	3	2	2
PVT1	29,510,227	4	3	4
GAS5	27,951,730	5	5	5
NEAT1	27,446,393	6	10	8
FENDRR	Unknown	7	13	14
UHRF1	Unknown	8	59	69
TUG1	29,654,398	9	6	6
XIST	7,981,672	10	7	9
KCNQ1OT1	Unknown	18	8	17
CCAT1	Unknown	14	9	10
MIAT	Unknown	12	12	7

Moreover, lung cancer is also a leading cause of cancer death all over the world, regardless of gender [34]. FVTLDA_MLR and FVTLDA_ANN can successfully predict 8 and 7 confirmed lncRNAs out of the top 10 candidate lncRNAs respectively (Table 4). However, KATZLDA can only predict 1 confirmed lncRNAs out of the top 10 candidate lncRNAs. Additionally, the case study contrast scores of FVTLDA_MLR, FVTLDA_ANN and KATZLDA are 0.8670, 0.7414 and 0.0998 respectively.

**Table 4:** Top 10 potential lung cancer-related lncRNAs and their PubMed unique identifiers predicted by FVTLDA_MLR, FVTLDA_ANN and KATZLDA

lncRNA	Evidence (PMID)	Rank by FVTLDA_MLR	Rank by FVTLDA_ANN	Rank by KATZLDA
PVT1	28,731,781	1	1	4
TUG1	28,069,000; 29,277,771	2	2	45
NEAT1	25,818,739	3	5	51
HOTTIP	26,265,284	4	6	49
XIST	Unknown	5	3	52
DANCR	29,651,883	6	11	63
MIAT	29,487,526	7	10	20
KCNQ1OT1	27,222,340	8	4	60
MIR155HG	Unknown	9	7	58
TP53TG1	Unknown	10	8	17
HOXA11-AS	29,616,096	20	9	54
DLX6-AS1	Unknown	61	61	1
LINC00511	Unknown	62	49	2
GNAS-AS1	Unknown	20	43	3
HCG11	Unknown	50	55	5
LINC00342	Unknown	58	51	6
MIR17HG	Unknown	47	62	7
SBF2-AS1	Unknown	33	25	8
SNHG12	Unknown	46	50	9
HCP5	Unknown	51	22	10

In conclusion, FVTLDA can achieve excellent prediction performance, and the average case study contrast scores of FVTLDA_MLR (0.8429) and FVTLDA_ANN (0.8515) are both higher than KATZ (0.6375).

## Discussion

A lot of evidence has demonstrated that lncRNAs play an important role in the pathological changes of human diseases, and identification of disease-related lncRNAs can help us better understand the disease mechanisms at the molecular level. However, it is costly and time-consuming to verify lncRNA-disease associations with biological experiments. Thus, it is important and necessary to develop efficient computational models to predict potential lncRNA-disease associations.

Different from state-of-the-art prediction models, in this paper, a novel computational model called FVTLDA is proposed to predict potential lncRNA-disease associations based on direct and indirect biological information. In order to avoid the limitation of the single model prediction technique, we further combine FVTLDA with multiple linear regression and artificial neural networks respectively. Moreover, to evaluate the prediction performance of FVTLDA, we conducted intensive in experiments. Simulation results demonstrate that FVTLDA achieves better performance than other six available state-of-the-art prediction models. Additionally, in case studies of gastric cancer, leukemia and lung cancer, simulation results show that the prediction ability and stability of both FVTLDA with MLR and FVTLDA with ANN are better than that of competing methods.

Certainly, despite the prediction performance of FVTLDA, the current version of FVTLDA can further improve performance as well. For example, we can increase the complexity of neural networks in the model of FVTLDA. Finally, more useful information sources including the gene-disease associations can be integrated into the feature vectors of lncRNA-disease pairs to further improve the prediction performance of FVTLDA. In the future, we can also study the association prediction in various fields of computational biology, such as miRNA-disease association prediction [35–37], drug-target interaction prediction [38, 39], and then bring valuable insights to the development of lncRNA-disease association prediction.

## Conclusion

In this manuscript, a novel computational model named FVTLDA is proposed. FVTLDA solved three problems of other models: (1) Some models can not be applied to isolated nodes. (2) Some methods require negative samples that are difficult to obtain. (3) Some approaches may be biased towards known nodes. Besides, we combine FVTLDA with Multiple Linear Regression and Artificial Neural Network for data analysis respectively, and results and case studies show that our model outperforms other state-of-the-art models, which indicate that FVTLDA can be an excellent tool for research in the future.

## Method

In order to introduce direct and indirect biological information on lncRNA-disease associations into FVTLDA, in this section, we first collected three kinds of known associations including miRNA-disease associations, miRNA-lncRNA associations and lncRNA-disease association from various databases. And then, based on these three kinds of datasets, we constructed three kinds of incidence matrix as follows:

*Step 1* First, we downloaded the dataset of known miRNA-disease associations and miRNA-lncRNA associations from the databases of HMDD [40] and starBase v2.0 [41] respectively. After having removed the repetitive associations supported by multiple evidences, and normalized the names of the miRNAs in these two datasets, we finally obtained 4704 unique miRNA-disease associations between 246 miRNAs and 373 diseases (see Additional file 6), and 9086 different miRNA-lncRNA association between 246 miRNAs and 1089 lncRNAs (see Additional file 7). Thereafter, based on these two datasets, we constructed a 246 × 373 dimensional miRNA-disease association incidence matrix *MD* and a 246 × 1089 dimensional miRNA-lncRNA association incidence matrix *ML* separately. In *MD*, there is *MD*(*i*,*j*) = 1, if and only if there exists a known association between the miRNA *m*_*i*_ and the disease *d*_*j*_, otherwise there is *MD*(*i*,*j*) = 0. Similarly, in *ML*, there is *ML*(*i*,*j*) = 1, if and only if there exists a known association between the miRNA *m*_*i*_ and the lncRNA *l*_*j*_, otherwise there is *ML*(*i*,*j*) = 0. For convenience, we defined the numbers of miRNAs, diseases and lncRNAs obtained above as *N*_*m*_, *N*_*d_MD*_ and *N*_*l_ML*_ respectively. Obviously, there are *N*_*m*_ = 246, *N*_*d_MD*_ = 373 and *N*_*l_ML*_ = 1089.

**Fig. 6:**
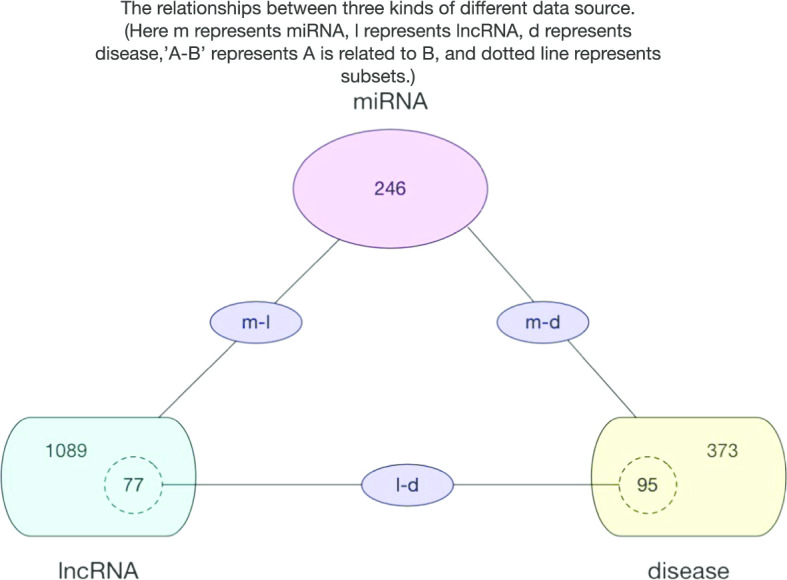
The relationships between three kinds of different data sources

*Step 2* Next, we downloaded the dataset of known lncRNA-disease associations from the MNDR v2.0 database [42]. After having removed the duplicate associations with multiple evidence, as illustrated in the Fig. 6, we further got rid of these associations with either lncRNAs not belonging to *N*_*l_ML*_ or diseases not belonging to *N*_*d_MD*_. Finally, we obtained 407 lncRNA-disease associations between 77 different lncRNAs and 95 different diseases (see Additional file 8). similarly, based on the newly-downloaded dataset, we constructed a 77 × 95 dimensional lncRNA-disease association incidence matrix *LD*, in which, there is *LD*(*i*,*j*) = 1, if and only if there exists a known association between the lncRNA *l*_*i*_ and the disease *d*_*j*_, otherwise there is *LD*(*i*,*j*) = 0. And for convenience, we define the numbers of lncRNAs and diseases obtained above as *N*_*l_LD*_ and *N*_*d_LD*_ respectively. Obviously, there are *N*_*l_LD*_ = 77 and *N*_*d_LD*_ = 95.

### Construction of the Gaussian interaction profile kernel similarity for miRNAs based on miRNA-lncRNA associated information

According to the assumption that similar miRNAs tend to interact with similar lncRNAs [43], the Gaussian interaction profile kernel similarity between the miRNA *m*_*i*_ and the miRNA *m*_*j*_ can be calculated as follows:

4 $$KM\left( {m_{i} ,m_{j} } \right) = \exp \left( { - \gamma_{m} \left| {\left| {IP\left( {m_{i} } \right) - IP\left( {m_{j} } \right)} \right|} \right|^{2} } \right)$$

5 $$\gamma_{m} = \frac{{\gamma_{m}^{\prime } }}{{\mathop \sum \nolimits_{k = 1}^{{N_{m} }} \left| {\left| {IP\left( {m_{k} } \right)} \right|} \right|^{2} }}$$

Here, *IP(m*_*i*_*)* denotes the *i*th row in the miRNA-lncRNA association incidence matrix *ML*, *γ*_*m*_ denotes the normalized bandwidth based on the new bandwidth parameter *γ*_*m*_′, and in this paper γ_m_′ will be set to 1 according to previous experiments [44]. In this way, an *N*_*m*_ × *N*_*m*_ dimensional Gaussian interaction profile kernel similarity matrix *KM* for miRNAs can be established.

### Construction of the functional similarity for miRNAs based on miRNA-disease associated information

In recent years, disease semantic similarity has been widely utilized to identify potential miRNA-disease associations, and many previous researches have shown the validity of this similarity [45–50]. In this study, we calculated the disease semantic similarity in the same way as in previous studies [49]. For all diseases, we first downloaded its corresponding Medical Subject Headings (MESH) descriptors from the National Library of Medicine in turn (http://www.nlm.nih.gov/) [49], and then, we represent a disease *d*_*A*_ as its directed acyclic graph (DAG) such as DAG(*d*_*A*_) = (*D*(*d*_*A*_), *E*(*d*_*A*_)). Here, *D*(*d*_*A*_) consists of the disease node *d*_*A*_ itself and all ancestor nodes of *d*_*A*_, while *E*(*d*_*A*_) is composed of all the directed edges from parent nodes to children nodes. For example, the code for breast neoplasm is: c04.588.180; c17.800.090.500. The corresponding parent nodes are C04.588 neoplasms by site and C17.800.090 breast diseases [49]. In the same way of the previous study [18], for any two disease nodes *d* and *t*, we will calculate the contribution of *t* to the semantic value of *d* as follows:

6 $$D_{d} \left( t \right) = \left\{ {\begin{array}{*{20}l} 0 \hfill & {if\;t \notin DAG\left( d \right)} \hfill \\ 1 \hfill & { if\;t \in DAG\left( d \right) \;and\; t = d} \hfill \\ {\max \left\{ {\Delta *D_{d} \left( {t^{\prime}} \right){|}t^{\prime} \in children of t} \right\}} \hfill & {if\; t \in DAG\left( d \right)\; and\; t \ne d} \hfill \\ \end{array} } \right.$$

where *∆* denotes the semantic contribution decay factor, and according to the previous study [49], in this paper, *∆* will be set to 0.5. Thereafter, we can calculate the semantic value of the disease *d* through combining all these diseases in its DAG(*d*) as follows:

7 $$D\left( d \right) = \mathop \sum \limits_{{t_{i} \in DAG\left( d \right)}} D_{d} \left( {t_{i} } \right)$$

According to the assumption that two diseases with a larger number of shared nodes in their DAGs may have higher similarity, we can calculate the disease semantic similarity score between a pair of diseases *d*_*i*_ and *d*_*j*_ as follows:

8 $$DS_{MD} \left( {i,j} \right) = \frac{{\mathop \sum \nolimits_{{t \in \left( {DAG\left( {d_{i} } \right) \cap DAG\left( {d_{j} } \right)} \right)}} \left( {D_{{d_{i} }} \left( t \right) + D_{{d_{j} }} \left( t \right)} \right)}}{{D\left( {d_{i} } \right) + D\left( {d_{j} } \right)}}$$

According to the above formula, it is obvious that an *N*_*d_MD*_ × *N*_*d_MD*_ dimensional matrix *DS*_*MD*_ can be established. Meanwhile, after extracting the semantic similarity information of disease in the lncRNA-disease association from the matrix *DS*_*MD*_, we can further build an *N*_*d_LD*_ × *N*_*d_LD*_ dimensional matrix *DS*_*LD*_ as well.

Apparently, after obtaining the semantic similarity scores of diseases, we can finally obtain the functional similarity between miRNAs based on the assumption that miRNAs with similar functions are often implicated in similar disease [49] as follows: for any two given miRNAs *m*_*i*_ and *m*_*j*_, let all diseases known to be related to *m*_*i*_ and *m*_*j*_ be *GDM*(*m*_*i*_) = {*d*_*i1*_,*d*_*i2*_,*d*_*i3*_…,*d*_*ip*_} and *GDM*(*m*_*j*_) = {*d*_*j1*_,*d*_*j2*_,*d*_*j3*_,…,*d*_*jq*_} respectively, then the functional similarity score between *m*_*i*_ and *m*_*j*_ can be calculated according to the following:

9 $$FM\left( {m_{i} ,m_{j} } \right) = \frac{{\mathop \sum \nolimits_{t = 1}^{p} \max \left( {DS_{MD} \left( {d_{it} ,GDM\left( {m_{j} } \right)} \right)} \right) + \mathop \sum \nolimits_{t = 1}^{q} {\max}\left( {DS_{MD} \left( {d_{jt} ,GDM\left( {m_{i} } \right)} \right)} \right)}}{p + q}$$

According to the above equation, an *N*_*m*_ × *N*_*m*_ dimensional functional similarity matrix *FM* for miRNAs can be established. In the same way, let all diseases are known to be associated to lncRNAs *l*_*i*_ and *l*_*j*_ as *GDL*(*l*_*i*_) = {*d*_*i1*_,*d*_*i2*_,*d*_*i3*_…,*d*_*ip*_} and *GDL*(*l*_*j*_) = {*d*_*j1*_,*d*_*j2*_,*d*_*j3*_,…,*d*_*jq*_} separately, then the functional similarity score between *l*_*i*_ and *l*_*j*_ can as well be calculated according to the following equation:

10 $$FL\left( {l_{i} ,l_{j} } \right) = \frac{{\mathop \sum \nolimits_{t = 1}^{p} \max \left( {DS_{LD} \left( {d_{it} ,GDL\left( {m_{j} } \right)} \right)} \right) + \mathop \sum \nolimits_{t = 1}^{q} {\max}\left( {DS_{LD} \left( {d_{jt} ,GDL\left( {m_{i} } \right)} \right)} \right)}}{p + q}$$

### Construction of FVTLDA

**Fig. 7:**
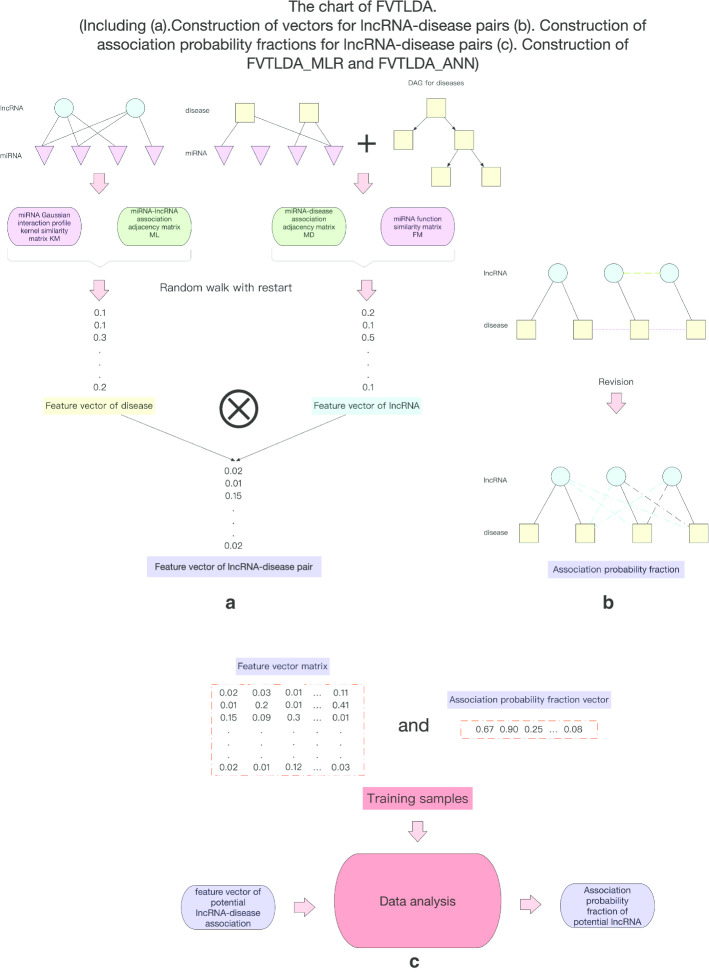
The flowchart of FVTLDA

As illustrating in Fig. 7, FVTLDA consists of the following three major steps:

*Step a* According to indirect biological information including known miRNA-lncRNA associations and known miRNA-disease associations downloaded above, for each pair of lncRNA and disease, a unique feature vector will be constructed first by adopting the random walk with restart based on the Gaussian interaction profile kernel similarity for miRNAs and functional similarity for miRNAs.

*Step b* Next, according to known lncRNA-disease associations downloaded above, for each pair of lncRNA and disease, a unique association probability fraction will be calculated based on the concept of Disease Clique [25].

*Step c* Finally, based on the feature vectors and association probability fractions obtained above, the Multiple Linear Regression (MLR) and the Artificial Neural Network (ANN) will be integrated to infer relationships between feature vectors and corresponding association probability fractions. And then, based on these predicted relationships, for each pair of lncRNA and disease, the potential association between them will be mapped into a probability score. Thereafter, based on these probability scores, we can rank the associations between lncRNAs and diseases conveniently.

#### Construction of feature vectors for lncRNA-disease pairs

As showing in Fig. 7a, for each lncRNA-disease pair, the construction of its feature vector consists of the three major steps:

*Step 1* Based on the formula (11), construct the miRNA-lncRNA association probability fractions matrix *PL* according to known miRNA-lncRNA associations and the Gaussian interaction profile kernel similarity for miRNAs. And then, for each lncRNA *l*_*i*_, the column corresponding to *l*_*i*_ in the matrix *PL* will be considered as the feature vector of *l*_*i*_.

*Step 2* Based on the formula (14), construct miRNA-disease association probability fractions matrix *PD* according to known miRNA-disease associations and the miRNA functional similarity. And then, for each disease *d*_*j*_, the column corresponding to *d*_*j*_ in the matrix *PD* will be considered as the feature vector of *d*_*j*_.

*Step 3* For each lncRNA-disease pair (*l*_*i*_,*d*_*j*_), obtain its feature vector through integrating the feature vector of *l*_*i*_ with the feature vector of *d*_*j*_ according to the following formula (17).

Random Walk is usually adopted to sort the association probabilities of nodes in a network [50], therefore we can implement the random walk with restart on the miRNA-lncRNA association network to obtain the feature vector of lncRNAs as follows: Let any given lncRNA node *l*_*i*_ as the walker, the random walks will start from all known miRNA nodes related to it, and will be moved from the current node to the next node according to the Gaussian interaction profile kernel similarity for miRNA nodes. During implementing the random walk, supposing that the random walk can be restarted with the probability of *r*_*1*_ (0 < *r*_*1*_ < 1), then the random walk process can be described by the following formulas:

11 $$PL_{s + 1} = \left( {1 - r_{1} } \right)*NKM^{T} *PL_{s} + r_{1} *PL_{0}$$

12 $$NKM\left( {i,j} \right) = \frac{{KM\left( {i,j} \right)}}{{\mathop \sum \nolimits_{k = 1}^{{N_{m} }} KM\left( {i,k} \right)}}$$

13 $$PL_{0} \left( {i,j} \right) = \frac{{ML\left( {i,j} \right)}}{{\mathop \sum \nolimits_{k = 1}^{{N_{m} }} ML\left( {k,j} \right)}}$$

The random walk process is an iterative process, which will be stopped when the random walk reaches a stable state: Here, considering the requirements of time efficiency and accuracy, the random walk will be considered to be stable if the difference between *PL*_*s*+*1*_ and *PL*_*s*_ is less than 10^–10^. In this way, for each lncRNA *l*_*i*_, it is obvious that the feature vector of *l*_*i*_ can be expressed by the association probability fractions of all miRNAs related to *l*_*i*_, i.e., the feature vectors of *l*_*i*_ can be expressed by the *i*th column in the matrix *PL*.

Similarly, for each disease d_j_, let the random walk be restarted with the probability of *r*_*2*_ (0 < *r*_*2*_ < 1), and its feature vector can as well be obtained according to the following equations:

14 $$PD_{s + 1} = \left( {1 - r_{2} } \right) * NFM^{T} * PD_{s} + r_{2} * PD_{0}$$

15 $$NFM\left( {i,j} \right) = \frac{{FM\left( {i,j} \right)}}{{\mathop \sum \nolimits_{k = 1}^{{N_{m} }} FM\left( {i,k} \right)}}$$

16 $$PD_{0} \left( {i,j} \right) = \frac{{MD\left( {i,j} \right)}}{{\mathop \sum \nolimits_{k = 1}^{{N_{m} }} MD\left( {k,j} \right)}}$$

Finally, for each lncRNA-disease pair (*l*_*i*_,*d*_*j*_), its feature vector can be calculated by combining the feature vectors of both *l*_*i*_ and *d*_*j*_ as follows:

17 $$FV_{ij} = PL\left( i \right) \otimes PD\left( j \right)$$

Here, *PL*(*i*) and *PD*(*j*) represent the *i*th column of the matrix *PL* and *j*th column of the matrix *PD* respectively. Moreover, for two column vectors A = (a_1_, a_2_,…,a_n_)^T^ and B = (b_1_,b_2_,…,b_n_)^T^, A $\otimes$ B = (a_1_ × b_1_,a_2_ × b_2_,…,a_n_ × b_n_)^T^.

In this way, all the feature vector obtained will be independent and there is no collinearity.

#### Construction of association probability fractions for LncRNA-disease pair

**Fig. 8:**
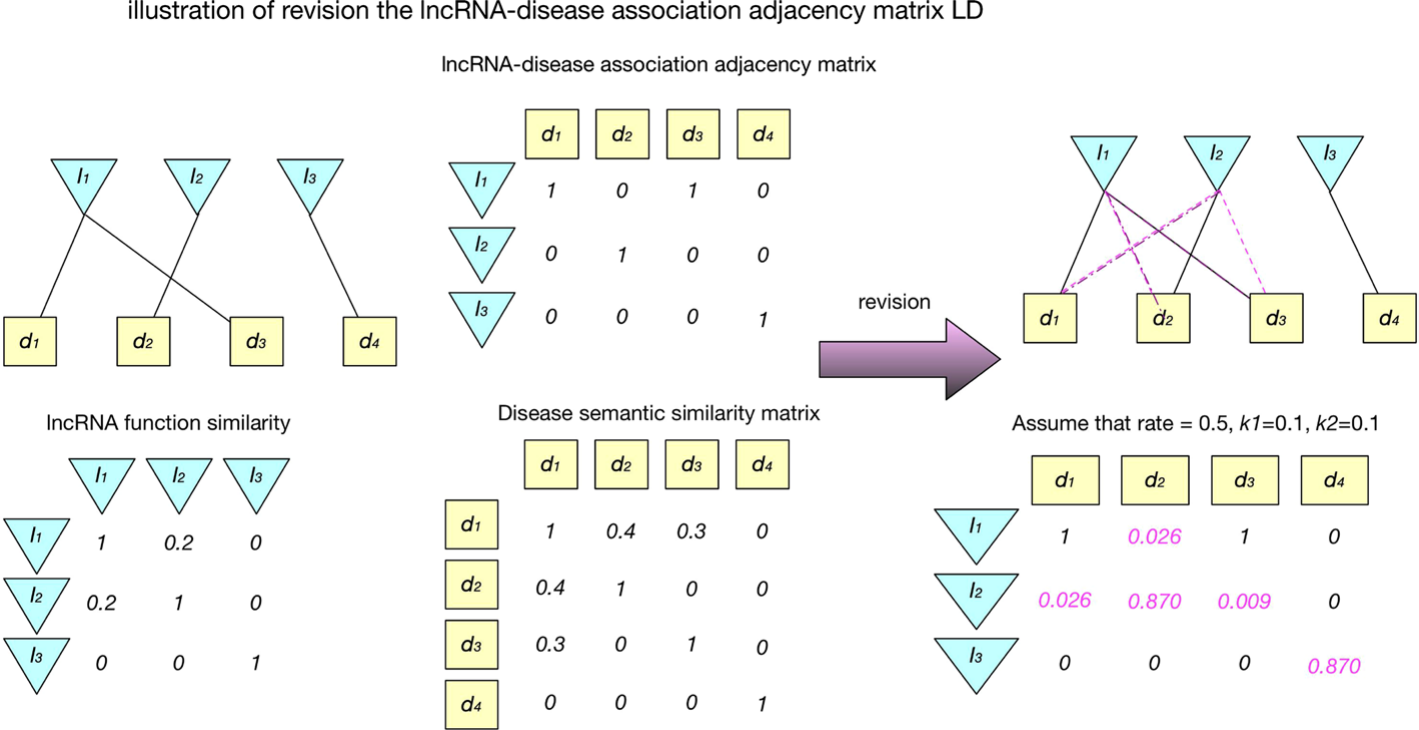
Constructing the probability fraction matrix *OUTPUT* based on *LD*

The incidence matrix *LD* obtained from known lncRNA-disease associations can only reflect whether or not lncRNAs have known associations with diseases, but cannot accurately express the degrees of their relationships. Moreover, if one element in *LD* equals 0, it only means that there is currently no known association between the pair of the corresponding lncRNA and disease nodes, but does not mean that there is absolutely no association existing between them. Thus, values in the matrix *LD* need to be further processed. Here, we turn this classification problem into a regression problem. By referring to the definition of the Disease Clique proposed in previous study [25], in this section, for each given disease *d*_*i*_ and lncRNA *l*_*j*_, we define the set consisting of all these nonzero elements in the *i*th row of the matrix *DS*_*LD*_ as the Disease Clique of *d*_*i.*_ Then, as shown in Fig. 8, the lncRNA-disease association incidence matrix *LD* can be revised as follows:

18 $$OUTPUT\left( {i,j} \right) = \frac{{OUT\left( {i,j} \right) - {\min}\left( {OUT} \right)}}{{\max \left( {OUT} \right) - {\min}\left( {OUT} \right)}}$$

19 $$OUT = rate * FOUT + \left( {1 - rate} \right) * DOUT$$

20 $$FOUT\left( {i,j} \right) = \left\{ {\begin{array}{*{20}l} {\mathop \sum \limits_{n = 1}^{{N_{l\_LD} }} k_{1} * LD\left( {n,j} \right) * FL\left( {i,n} \right)} \hfill & {if\;LD\left( {i,j} \right) \ne 1} \hfill \\ {\left( {\mathop \sum \limits_{n = 1}^{{N_{l\_LD} }} k_{1} * LD\left( {n,j} \right) * FL\left( {i,n} \right)} \right) + 1 - k_{1} } \hfill & {if\;LD\left( {i,j} \right) = 1} \hfill \\ \end{array} } \right.$$

21 $$DOUT\left( {i,j} \right) = \left\{ {\begin{array}{*{20}l} {\mathop \sum \limits_{n = 1}^{{N_{d\_LD} }} k_{2} * LD\left( {i,n} \right) * DS_{LD} \left( {n,j} \right)} \hfill & {if\;LD\left( {i,j} \right) \ne 1} \hfill \\ {\left( {\mathop \sum \limits_{n = 1}^{{N_{d\_LD} }} k_{2} * LD\left( {i,n} \right) * DS_{LD} \left( {n,j} \right)} \right) + 1 - k_{2} } \hfill & { if\;LD\left( {i,j} \right) = 1} \hfill \\ \end{array} } \right.$$

The probability fraction matrix *OUTPUT* obtained from the above formula (18) can not only solve the problem of sparsity existing in the original association incidence matrix *LD*, but also reflect the degree of relationship between lncRNAs and diseases to some extent.

#### Construction of FVTLDA with MLR and FVTLDA with ANN

In order to avoid the limitations of single model prediction scheme, for any given pair of lncRNA and disease nodes, in this section, we present two different methods, such as the Multiple linear regression (MLR) analysis and the Artificial neural network (ANN), to reveal the potential relationship between the feature vector of the lncRNA-disease pair and its association probability fraction.

##### Construction of FVTLDA with MLR

MLR analysis is often used in statistical analysis [51–53], whose purpose is to determine the quantitative relationship between the dependent and independent variables, and the general form of MLR can be expressed as follows:

22 $$Y = \beta_{0} + \beta_{1} X_{1} + \beta_{2} X_{2} + \cdots + \beta_{k} X_{k} \pm e$$

Here, *Y* represents the dependent variable, {*X*_*1*_,* X*_*2*_,…, *X*_*k*_} denote the independent variable of *Y*, *β*_*0*_ is the constant term, {*β*_*1*_, *β*_*2*_,…, *β*_*k*_} are the partial regression coefficients of {*X*_*1*_, *X*_*2*_,…,* X*_*k*_} respectively, and *e* denotes the error value. Based on formula (22), for each lncRNA-disease pair (*l*_*i*_,*d*_*j*_), we can represent the relationship between its association probability fraction *OUTPUT*(*i*,*j*) and its feature vector as follows:

23 $$OUTPUT\left( {i,j} \right) = \beta_{0} * 1 + \beta_{1} * FV_{ij} \left( 1 \right) + \beta_{2} * FV_{ij} \left( 2 \right) + \cdots + \beta_{{N_{m} }} * FV_{ij} \left( {N_{m} } \right)$$

Moreover, for convenience, we define the regression coefficients as W = [$\upbeta _{0}$,$\upbeta _{1}$,$\upbeta _{2}$,…,$\upbeta _{{{\text{N}}_{{\text{m}}} }}$], the feature vector of (*l*_*i*_,*d*_*j*_) as *x*_*n*_ = [1,*FV*_*ij*_(1),*FV*_*ij*_(2),…,*FV*_*ij*_(*N*_*m*_)], and the association probability fraction corresponding to(*l*_*i*_,*d*_*j*_) as *y*_*n*_ = *OUTPUT*(*i,j*)*.* Then, for a given training set *T* = {(*x*_1_,*y*_1_),(*x*_2_,*y*_2_),…,(*x*_*N*_,*y*_*N*_)}, let X = (x_1_,x_2_,…,x_n_)^T^ and Y = (y_1_,y_2_,…,y_n_)^T^, the regression coefficients *W* can be calculated by the least square method, and the optimal solution W^*^ can be calculated as follows:

24 $$W^{*} = \left( {X^{T} X} \right)^{ - 1} X^{T} Y$$

Finally, based on the above formulas, our prediction model FVTLDA with MLR can be described as the following Algorithm 1 (in Additional file 9).

##### Artificial neural network (ANN)

**Fig. 9:**
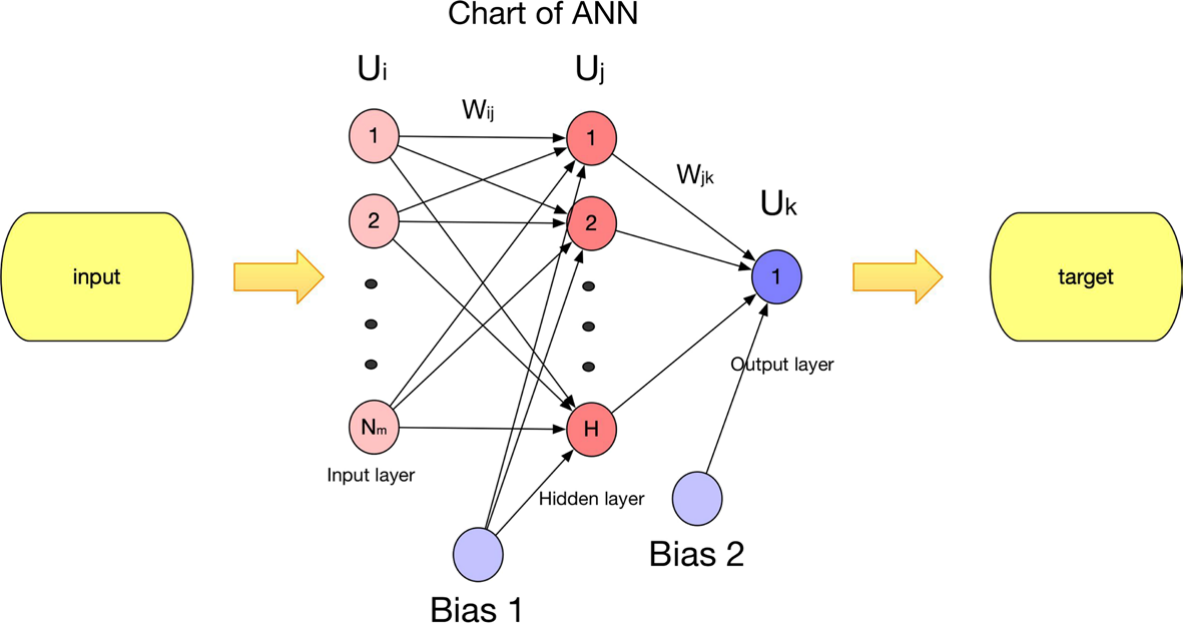
Chart of ANN

ANN is a simple model often used to simulate the biological structure of the human brain. It is a highly dense network composing of simple elements, which can reflect the essential relationships between dependent variables and independent variables. One of the most important characteristics of ANN is that it can be learned by training samples, which can overcome the limitations of traditional methods. Therefore, in this section, we will further adopt ANN to estimate the relationships between the feature vectors of lncRNA-disease pairs and their association probability fractions. As illustrating in the Fig. 9, ANN is a parallel distributed processing system composing of many process components (neurons), which can be divided into three layers such as the Input layer, the Hidden layer and the Output layer. In ANN, each neuron in every layer can receive one or more input signals, and generate an output signal through the activation function as the input signal of the next layer. The most important part of ANN is to determine the weights and biases. In ANN, each link between neurons represents a weight that reflects the influence of the previous neuron on the current neuron, and bias can increase the flexibility of this neuron [54]. In this section, in a way similar to the previous study [55], we determine the weights and biases of ANN through the following four major steps:

*Step 1* Take the training samples as the input values, and randomly set the initial values of weights and biases in each layer of ANN.

*Step 2* Calculate the output of ANN and compare the output with the target value to obtain the value of error.

*Step 3* Readjust the weights and biases in each layer of ANN according to the value of error obtained above from Step 2.

*Step 4* Repeat the above procedure until ANN reaches the stop condition.

In this paper, all feature vectors of lncRNA-disease pairs were randomly divided into the training set, the validation set and the test set in a ratio of 3:1:1. Moreover, the training sets were taken as the input of the Input layer. Thereafter, the input of the Hidden layer can be obtained by combining the weights, the output of the Input layer and the biases. Additionally, let $I_{m}^{n}$ and $O_{m}^{n}$ denote the input value and the output value of the node *m* in the *n*th layer of ANN separately, then, the output of the Hidden layer can be calculated according to the following activation function:

25 $$O_{X}^{2} = \frac{2}{{1 + e^{{ - 2*I_{X}^{2} }} }} - 1$$

Similarly, the input of the Output layer can be acquired by integrating the weights and the output of the Hidden layer, and the output of the Output layer can be figured out through the following activation function:

26 $$O_{1}^{3} = I_{1}^{3}$$

After obtaining the output value of the Output layer of ANN, the mean square error (MSE) can be obtained by comparing it with the target (the corresponding association probability fraction) as follows:

27 $$E_{total} = \frac{1}{N}\mathop \sum \limits_{k = 1}^{N} \left( {O_{1}^{3} \left( k \right) - target\left( k \right)} \right)^{2}$$

Here, *N* represents the number of test sets.

Finally, the weight and bias between each pair of neuron connections can be modified repeatedly according to the MSE value until one of the following stop conditions has been satisfied:

1. Maximum training times (were set to 100 in this paper)
2. Minimum MSE (was set to 0.001 in this paper)
3. Maximum times of consecutive iterations (In the training process, since the MSE of validation set does not decrease in *t* consecutive iterations, then we were set the maximum times of consecutive iterations to 15 in this paper)

Finally, based on the above formulas, our prediction model FVTLDA with ANN can be described as the following Algorithm 2 (in Additional file 9).

###### Availability and requirements

Project name: My bioinformatics project FVTLDA.

Project home page: https://github.com/xiaoyubin123/FVTLDA.git

Operating system: Platform independent

Programming language: Matlab

Other requirements: Matlab_R2017b or higher

Any restrictions to use by non-academics: No license required

## Supplementary Information

**Additional file 1** The ROC curves achieved by FVTLDA_ANN in framework of LOOCV.

**Additional file 2** The ROC curves achieved by FVTLDA_MLR in framework of 5-fold CV.

**Additional file 3** The ROC curves achieved by FVTLDA_MLR in framework of 10-fold CV.

**Additional file 4** The ROC curves achieved by FVTLDA_ANN in framework of 5-fold CV.

**Additional file 5** The ROC curves achieved by FVTLDA_ANN in framework of 10-fold CV.

**Additional file 6** Known miRNA-disease associations obtained from HMDD.

**Additional file 7** Known miRNA-lncRNA associations obtained from starBase v2.0.

**Additional file 8** Known lncRNA-disease associations obtained from MNDR v2.0.

**Additional file 9** Algorithm 1 and 2.

## Data Availability

All data generated or analyzed during this study are included in this published article [Additional files 6, 7 and 8].

## Abbreviations

FVTLDA: Feature vectors is developed to predict LncRNA-Disease Associations
LOOCV: Leave-one out cross validation
MLR: Multiple linear regression
ANN: Artificial neural network
CV: Cross validation
RWR: Random walk with restart
TPR: True positive rate
FPR: False positive rate
ROC: Receiver operating characteristic
AUC: Areas under ROC curve
