# Supplementary figures and images for: A novel computational model for predicting potential LncRNA-disease associations based on both direct and indirect features of LncRNA-disease pairs
Source: BMC Bioinformatics. 2020 Dec 2;21:555. doi: 10.1186/s12859-020-03906-7 (PMC7709313; doi:10.1186/s12859-020-03906-7)

Repeat the model 20 times in LOOCV framework

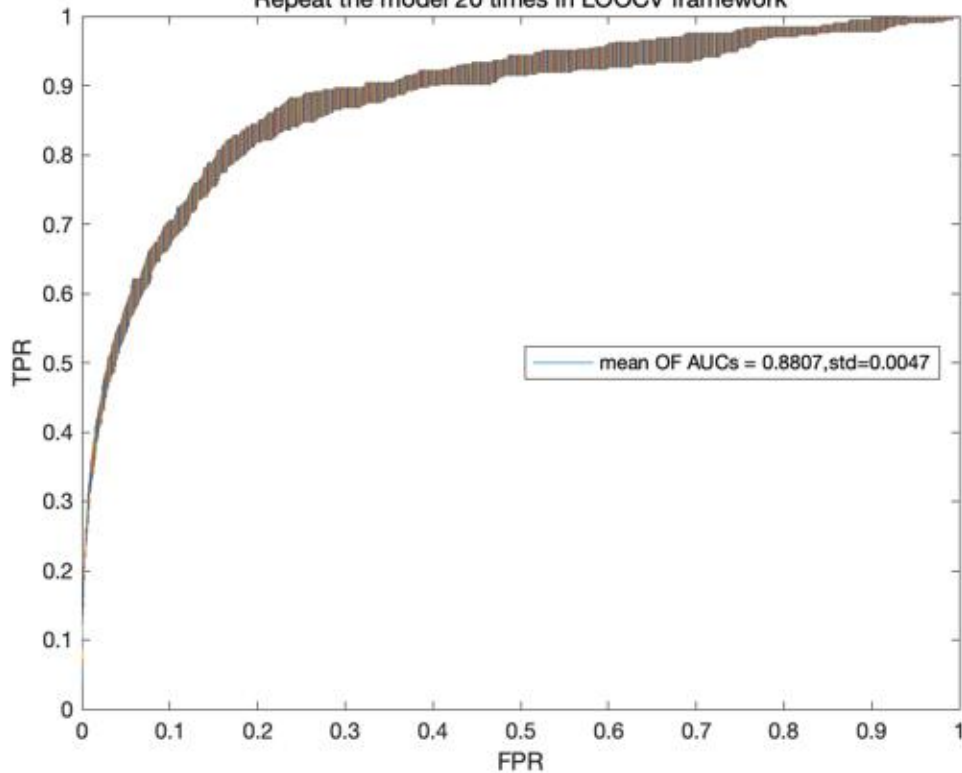

Supplement: Supplementary file 1 — Additional file 1 The ROC curves achieved by FVTLDA_ANN in framework of LOOCV. [file 12859_2020_3906_MOESM1_ESM.pdf]

Repeat the model 100 times in 5-CV framework

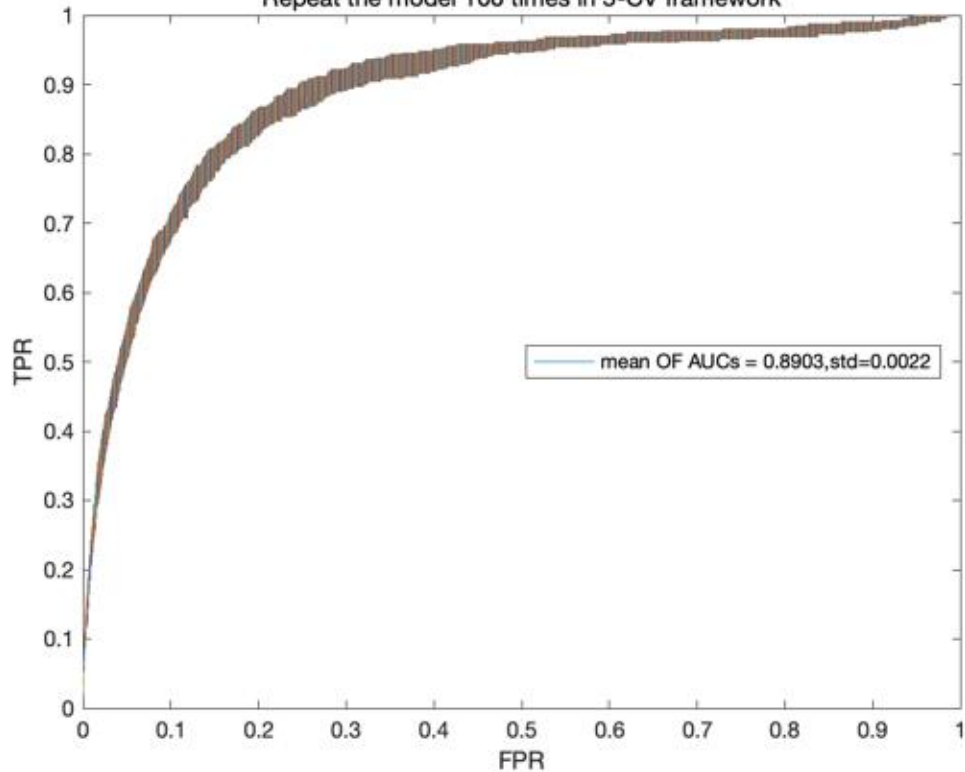

Supplement: Supplementary file 2 — Additional file 2 The ROC curves achieved by FVTLDA_MLR in framework of 5-fold CV. [file 12859_2020_3906_MOESM2_ESM.pdf]

Repeat the model 100 times in 10-CV framework

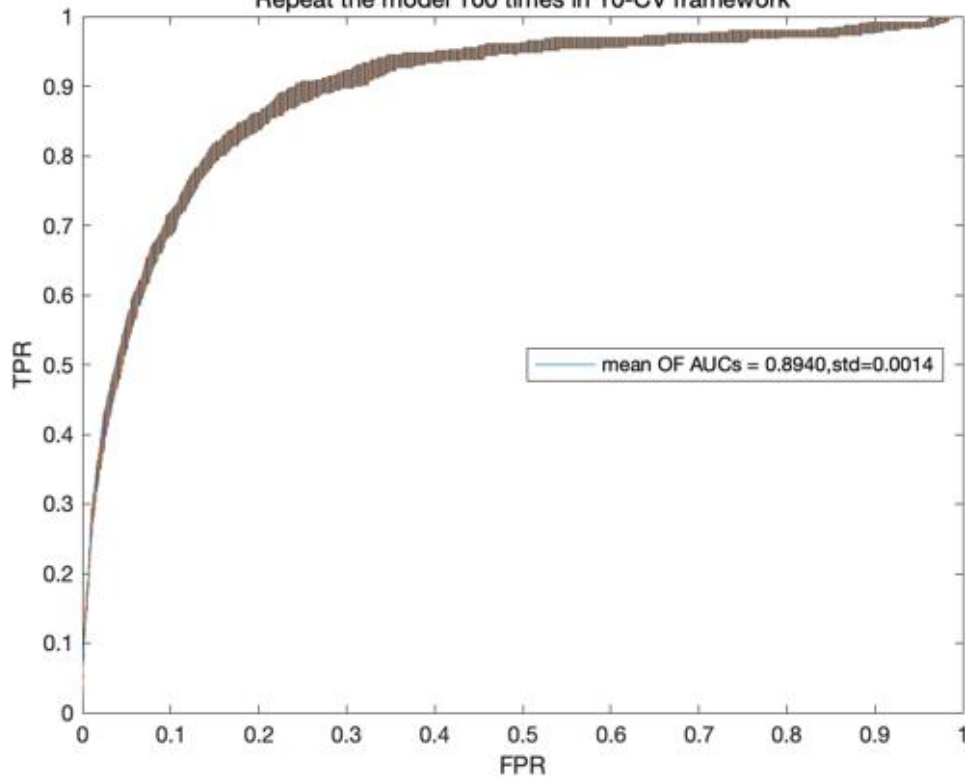

Supplement: Supplementary file 3 — Additional file 3 The ROC curves achieved by FVTLDA_MLR in framework of 10-fold CV. [file 12859_2020_3906_MOESM3_ESM.pdf]

Repeat the model 20 times in 5-CV framework

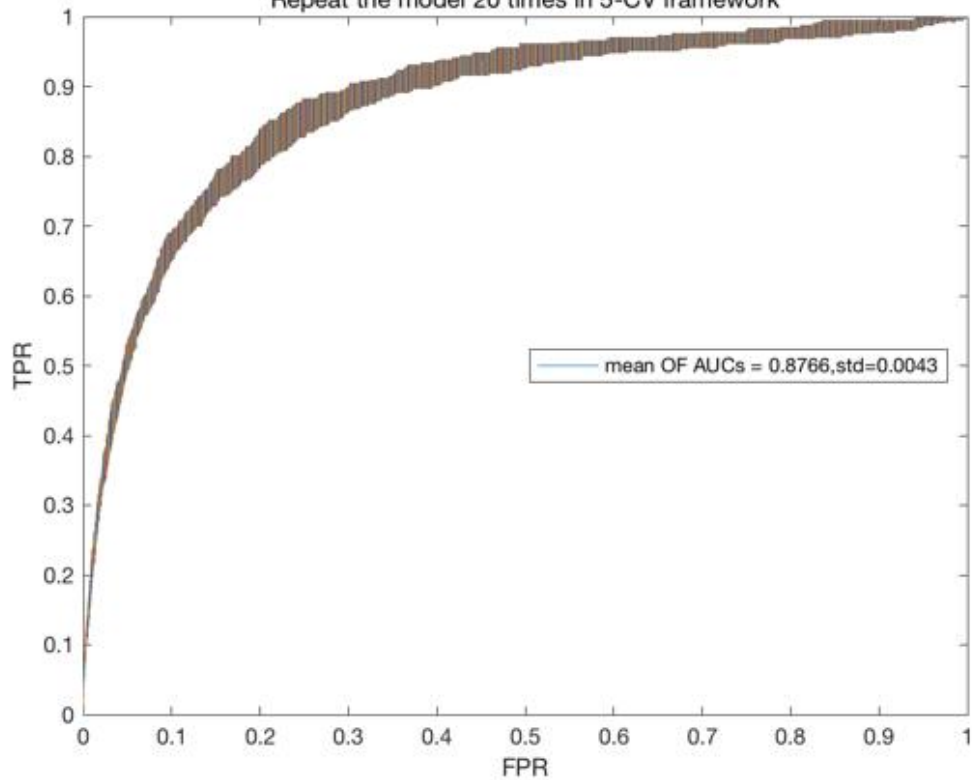

Supplement: Supplementary file 4 — Additional file 4 The ROC curves achieved by FVTLDA_ANN in framework of 5-fold CV. [file 12859_2020_3906_MOESM4_ESM.pdf]

Repeat the model 20 times in 10-CV framework

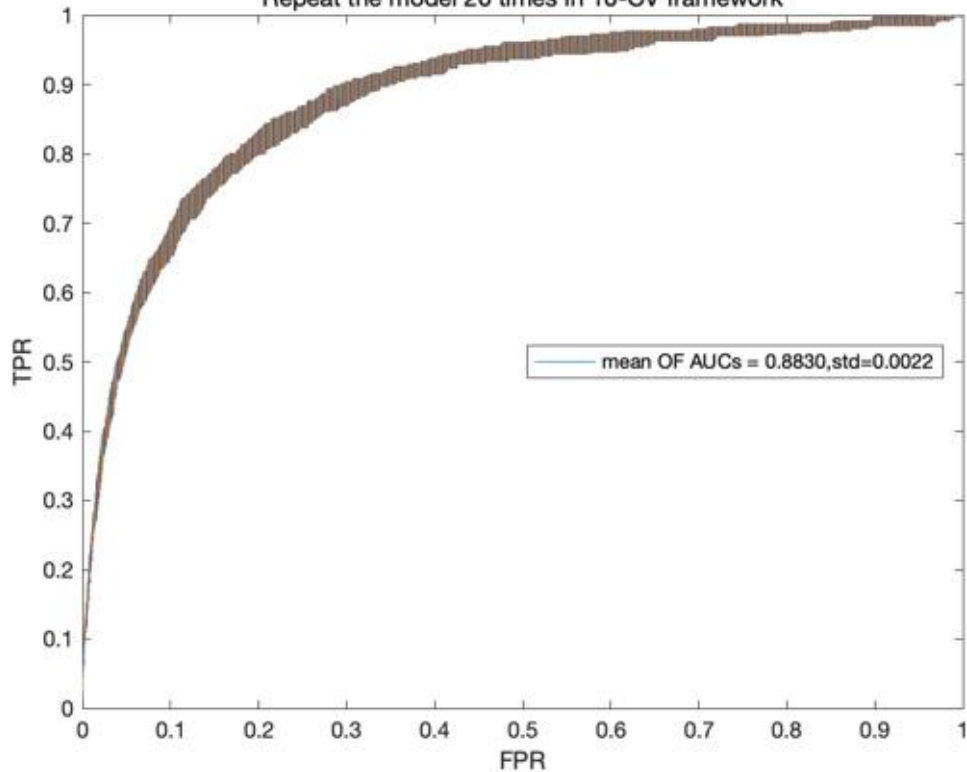

Supplement: Supplementary file 5 — Additional file 5 The ROC curves achieved by FVTLDA_ANN in framework of 10-fold CV. [file 12859_2020_3906_MOESM5_ESM.pdf]
