# Supplementary material for: A novel computational model for predicting potential LncRNA-disease associations based on both direct and indirect features of LncRNA-disease pairs
Source: BMC Bioinformatics. 2020 Dec 2;21:555. doi: 10.1186/s12859-020-03906-7 (PMC7709313; doi:10.1186/s12859-020-03906-7)
Supplement: Supplementary file 9 — Additional file 9 Algorithm 1 and 2. [file 12859_2020_3906_MOESM9_ESM.docx]

**Algorithm 2: FVTLDA with ANN**

**Input**: Known miRNA-lncRNA associations matrix *ML*, known miRNA-disease associations matrix *MD*, known lncRNA-disease associations matrix *LD*, disease MESH descriptors, parameter *r*_1_, *r*_2_, *k*_1_, *k*_2_, *rate*.

**Output**: The association probability fraction vector of candidate lncRNA-disease pairs.

**Step 1**: Generate the miRNA Gaussian interaction profile kernel similarity matrix KM by Eq (4);

**Step 2**: Generate the disease semantic similarity matrix DS_MD_ and DS_LD_ by Eq (8);

**Step 3**: Generate the miRNA function similarity matrix *FM* and lncRNA function similarity matrix *FL* by Eq (9) and Eq (10) respectively;

The training process of the FVTLDA with ANN is as follows:

**Step 4**: Generate feature vector *FV_ij_* for each pair of lncRNA *l_i_* and disease *d_j_* in the training set by Eq (17);

**Step 5**: Generate the association probability fraction *OUTPUT*(*i*,*j*) for each pair of lncRNA *l_i_* and disease *d_j_* in the training set by Eq (18);

**Step 6**: Take the *FV_ij_* as input values and *OUTPUT*(*i*,*j*) as the target values to generate the weights and biases of ANN according to the four major steps described above;

After obtaining weights and biases of ANN, the testing process of the FVTLDA with

ANN is as follows:

**Step 7**: Generate feature vector *FV_ij_* for each pair of lncRNA *l_i_* and disease *d_j_* in the testing set;

**Step 8**: Take the *FV_ij_* as input values to calculate the association probability fraction *score_ij_* for each pair of lncRNA *l_i_* and disease *d_j_* in the testing set;

(Here, score_ij_ is the output values of the ANN.)

**Step 9**: Sort all candidate lncRNA-disease pairs by the value of association probability fractions obtained by step 8 in the descending order;

**Step 10**: Output the sorted candidate lncRNA-disease pairs;

**Algorithm 1: FVTLDA with MLR**

**Input**: Known miRNA-lncRNA associations matrix *ML*, known miRNA-disease associations matrix *MD*, known lncRNA-disease associations matrix *LD*, disease MESH descriptors, parameter *r*_1_, *r*_2_, *k*_1_, *k*_2_, *rate*.

**Output**: The association probability fractions vector of candidate lncRNA-disease pairs.

**Step 1**: Generate the miRNA Gaussian interaction profile kernel similarity matrix KM by Eq (4);

**Step 2**: Generate the disease semantic similarity matrix DS_MD_ and DS_LD_ by Eq (8);

**Step 3**: Generate the miRNA function similarity matrix *FM* and lncRNA function similarity matrix *FL* by Eq (9) and Eq (10) respectively;

The training process of the FVTLDA with MLR is as follows:

**Step 4**: Generate feature vector *FV_ij_* for each pair of lncRNA *l_i_* and disease *d_j_* in the training set by Eq (17);

**Step 5**: Generate the association probability fractions *OUTPUT*(*i*,*j*) for each pair of lncRNA *l_i_* and disease *d_j_* in the training set by Eq (18);

**Step 6**: Obtain the optimal regression coefficients W∗ by Eq (24);

After obtaining W*, the testing process of the FVTLDA with MLR is as follows:

**Step 7**: Generate feature vector *FV_ij_* for each pair of lncRNA *l_i_* and disease *d_j_* in the testing set;

**Step 8**: Calculate the association probability fraction *score_ij_* for each pair of lncRNA *l_i_* and disease *d_j_* in the testing set as follows: *score_ij_* =*W*^*^×*FV_ij_* ;

**Step 9**: Sort all candidate lncRNA-disease pairs by the value of association probability fractions obtained by step 8 in the descending order;

**Step 10**: Output the sorted candidate lncRNA-disease pairs;
